# Research consent from young people in resource-poor settings

**DOI:** 10.1136/archdischild-2014-307121

**Published:** 2014-12-04

**Authors:** Phaik Yeong Cheah, Michael Parker

**Affiliations:** 1 Mahidol Oxford Tropical Medicine Research Unit, Faculty of Tropical Medicine, Mahidol University, Bangkok, Thailand; 2 Centre for Tropical Medicine, Nuffield Department of Medicine, University of Oxford, Oxford, UK; 3 Nuffield Department of Population Health, The Ethox Centre, University of Oxford, Oxford, UK

**Keywords:** Ethics, Adolescent Health, Tropical Paediatrics

## Abstract

Authoritative international guidelines stipulate that for minors to participate in research, consent must be obtained from their parents or guardians. Significant numbers of mature minors, particularly in low-income settings, are currently being ruled out of research participation because their parents are unavailable or refuse to provide consent despite the possibility that they might wish to do so and that such research has the potential to be of real benefit. These populations are under-represented in all types of clinical research. We propose that, for research with a prospect of direct benefit that has been approved by relevant ethics committees, the default position should be that minors who are able to provide valid consent and meet the following criteria should be able to consent for themselves regardless of age and whether they have reached majority: the minor must be competent and mature relative to the decision; their consent must be voluntary and they must be relatively independent and used to decision making of comparable complexity. In addition, the context must be appropriate, the information related to the research must be provided in a manner accessible to the minor and the consent must be obtained by a trained consent taker in surroundings conducive for decision making by the minor. In this paper, we have argued that consent by mature minors to research participation is acceptable in some situations and should be allowed.

## Introduction

Medical research involving children is both important and necessary.^1–3^ Research with children in low-income settings is particularly important as children make up a significant proportion of the population at risk of disease but are under-represented in all areas of clinical research. An important challenge is that in most countries, even if competent, potential paediatric study participants are dependent upon their parents or guardians to make decisions about their participation in clinical studies.^4^ ^5^ This poses challenges for researchers as some children may not live with their parents or have parents—they make important decisions in their daily lives, they are married and may be themselves parents. Although they are ‘emancipated’ minors socially, their status is not officially recognised by the law and research ethics committees. In these contexts, if parents are not available, these children are either excluded altogether or consent is sought from a ‘guardian’. The guardian can be the village elder, older sibling or employer, who may mean well but may not be fit surrogate decision makers.

Against this background we believe it is vital to think carefully about when, if ever, it might be acceptable for children to decide for themselves about research participation. It is our view that, on occasion, it is acceptable for children to make their own decision about whether to consent to participate in research with a prospect of direct benefit that has been approved by relevant ethics committees. In this paper, we explore these issues in relation to the following two hypothetical but realistic scenarios.

Scenario one: Soe Soe (SS) is 15 years old, married and works on a corn plantation in Myanmar. She is 4-months pregnant. One day, SS woke up with a fever and went to her village clinic to seek medical care. She walked for 2 hours to the clinic. The doctors told her that she had malaria and asked her if she wanted to participate in a randomised controlled trial. SS understood what the study was about and wanted to participate in the study. Her doctors told her that to participate one of her parents would have to provide consent on her behalf. To see her parents, SS would have to travel 2 days on foot.

Scenario two: A group of researchers are conducting a study in rural Bangladesh in which teenage children who had severe malaria as babies are assessed for long-term neurological impairment. Fatima is a bright 15-year-old girl who lives with her family. She goes to school in the morning and helps out in her mother's restaurant in the afternoon. A study field worker asked Fatima if she would like to participate in the study. Fatima understands what the study is about and says that she wants to participate in the study. The field worker asks Fatima's father to sign a consent form but he refuses. This means that Fatima cannot be enrolled in the study.

## Discussion

### Should minors be allowed to consent to research participation?

Situations such as those above are commonplace in low-income settings. They occur on a daily basis in most of our studies, at most of our sites. In both cases there is real scientific value in the minor's participation in the study. However, the current guidelines suggest that despite the minors’ wishes to participate neither of them be enrolled because their parents are unavailable or they refuse consent. Is this ethical? If we assume for the purposes of discussion that the studies are in all other respects ethically robust and approved by relevant ethics committee, should SS and Fatima be allowed to participate?

Based on our experience conducting research in low-income settings, we think that these situations are not uncommon and may be such that one or both should have been allowed to consent to participate in the study. In what follows we explore the arguments in favour of and against allowing children to consent to research participation and how they might apply to these two cases.

### Competence

Perhaps, the strongest argument for excluding children from decision making about research participation is that they tend to lack the competence to do so. However, this argument does not apply to children who *are* competent.^6^ ^7^ If children have the ability to meet the relevant criteria for competence—often described as involving the ability to understand and retain relevant information, to weigh or judge the relative merits of the options and to make and communicate a decision—they should be allowed to consent for themselves.^8^ Clearly in the two scenarios above, careful consideration would need to be given to the assessment of the competence of SS and Fatima but if after careful assessment they were judged to be competent, the ‘lack of competence’ argument against childhood consent to research would not be relevant. At least some and potentially many children are capable of meeting the competence threshold for valid consent.

### Voluntariness

An additional key requirement for valid consent is that such consent be voluntary; children such as SS and Fatima would need to have made their own free choice about whether to participate.

There are a number of ways in which consent might fail to be voluntary. In resource-poor settings, for example, the provision of healthcare in research projects can often act as an inducement. If participation in research was the only way for SS and Fatima to gain access to clinical care, this could potentially mean that their decision to participate was not voluntary in the required sense. Furthermore, there are a number of other ways in which the decisions of children might be coerced or less than ideally voluntary. They might be afraid of those in authority such as teachers, doctors and researchers, believing that they are not ‘allowed’ to refuse participation.

While these are all important considerations, careful assessment and judgement would be required about whether any particular competent minor was making a sufficiently voluntary decision. This is essentially an empirical question rather than a point of principle. If in a particular case a competent minor is capable of making a voluntary decision about research participation they should be allowed to do so.

### Maturity

One counterargument sometimes made against the claim that children can meet the relevant criteria for competence and voluntariness is that while they might appear to be of making a voluntary choice, they can often lack sufficient ‘maturity’ to make important decisions with long-term implications. The potential importance of maturity is acknowledged in many settings. For example, in the UK, children are allowed to consent to medical treatment if they are judged to have sufficient maturity.^9^ We agree that maturity may be relevant to some decisions about research participation and might on occasion mean that despite their competence in other respects, it would be inappropriate for a child to be allowed to consent in their own right.

If it is assumed for the purposes of discussion that both SS and Fatima are competent, we agree that it is also important to ask if they have sufficient maturity to make the decision at hand, the relevant level of maturity being relative to the significance of the decision. If the decision has the prospect of long-term consequences, then it is important to determine whether the minor has had the life experiences necessary to make such decisions. Genuine maturity, which is undeniably a challenge to assess,^10^ is a prerequisite for making decisions that are more significant in their consequences, involving perhaps substantial changes to a person's life prospects or where the decision may have irreversible effects. A great deal will depend on the nature of the study. If, as in Fatima's scenario, the study only involves assessments in an outpatient clinic with no impact on her future life or potential for irreversible side effects, then one might consider that she is sufficiently mature. On the other hand, if the interventions or procedures were more serious, for example, if SS's pregnancy might be at additional risk in a study involving a new drug with potentially serious side effects for the baby, then the threshold of maturity would appropriately be set higher. It is our view that, while judgement is required, children as a group should not be ruled out of participation in such research when many will have sufficient maturity to decide for themselves.

Assuming that, following a careful assessment, the study has been approved by relevant ethics committees, and that those recruiting participants are confident, the minor understands the implications of participation and has sufficient maturity to make a judgement about whether or not to participate, we think that it may be appropriate for a minor to make his or her own decision about whether to participate.

### The fixed-age threshold

While acknowledging many of the arguments above, some have argued that although there are some children who are competent and sufficiently mature, it makes pragmatic sense to have a single age threshold—the most common is 18 years of age.^11^ This is partly for resource reasons and partly for protection, and some believe that it is better to be safe than sorry. We disagree and see it as particularly problematic in situations where there is both a pressing need for research and there are a substantial number of children such SS and Fatima. In this context, there may be good reasons for devoting time and effort to identifying and assessing mature minors as research participants. The exclusion of mature minors from research participation can be both unfair and can have serious consequences. An example of this is a study on malaria in pregnancy in northwestern Thailand (trial registration: NCT01054248). Many pregnant teenagers missed the opportunity of being enrolled in the study as their parents were not available to consent on their behalf. These teenage mothers-to-be have their own families but the law and ethics committee guidelines are unclear about their provisions for these potentially emancipated minors.

### Additional protections

We have argued above that at least in some cases children who achieve the status of ‘mature minors’ ought to be able to make their own decisions about research participation. Having rejected the idea of a blanket ban on children's consent to research, we do believe that additional protections should be in place.

#### Independence

As a reasonable requirement for evidence of maturity, the minor should be relatively ‘independent’. This could mean having his or her own accommodation and job and the freedom to make decisions in daily life. This is also important for practical reasons where, for example, participants might need to make arrangements like travel to attend follow-up visits. While being wary about reading too much into our scenarios, this might suggest, for example, that because SS can go to the clinic herself and by contrast Fatima is living as a child in a family we might hypothesise that SS is likely to meet the independence criterion. The key point is that the minor should be sufficiently autonomous in their daily life to have to make decisions of comparable complexity as the decision that he/she is asked to make about research participation. In respecting the principle of autonomy, individuals who can shape their own lives should be allowed to decide whether to enrol in research in their own right.

#### Contextual appropriateness

The way doctors relate to minors in research should be consistent with how they relate to them in clinical care. Assuming that SS was offered the option of standard care at the clinic without requiring her parents’ consent, she should be allowed to consent for the research. This is being consistent. Contextual appropriateness also means that the decision making for participation in research should be consistent with decisions in daily life and reflects family dynamics. If Fatima normally gets her father's permission to do things, perhaps because she is a girl living in a relatively conservative society, then it is only consistent that she gets his permission to participate in the study. Understood more broadly, the requirement of contextual appropriateness suggests that children who are competently making decisions of comparable complexity, such as those being made by SS, ought to be able to make decisions about research participation. Clearly there are some children who are living ‘independently’ against their will and making decisions for which they are unprepared. Our proposal is not that all children living away from adult care are competent and mature. It is that where there is good evidence that this is the case, they ought to be able to make in their own right at least some decisions relating to participation.

#### Trained consent taker and accessible information

Another protection is that those who are requesting consent should have had appropriate training and should be aware of the challenges in obtaining valid consent from minors. They must have the skills to explain the information clearly, to assist the decision making and respect the potential participants’ decisions, without putting any undue pressure on them. In addition, they have to differentiate between a teenager who has the maturity to make a decision from one who has not and to assess whether the teenager has sufficient understanding of the pertinent issues. The consent discussion should be undertaken in appropriate surroundings, giving adequate time both for comprehension and for asking questions. Non-urgent decisions should be delayed until such a time as a minor's competence can be maximised. Information should be presented in ways and language that minors can understand and illustrated by meaningful examples and address concerns that are important to adolescents such as stigma and missing school. This can involve the use of videos, comics and other creative presentation of information.^12^

#### Lower age limit

Notwithstanding all of the above, we believe that to prevent any abuse, there should be a lower age threshold for consent. This age limit is thought to be between 12 and 14 years for most experimental research, but may be lower depending of the nature of the research.^3^

## Conclusion

In this paper, we have argued that consent by mature minors to research participation is acceptable in some situations and should be allowed. We have illustrated these situations using two case studies of under-aged girls. Our arguments should be no different for boys. In cases where the research is important, has the prospect of direct benefit, meets international ethical guidelines and has been approved by relevant ethics committees, children should sometimes be allowed to consent to participate in their own right, rather than being dependent upon the permission of an adult. We have set out the conditions and requirements for their consent to be deemed valid.

Significant numbers of mature minors are currently being ruled out of research participation because they have not reached the legal age of majority. We propose that the default position should be that minors who are able to provide valid consent and meet the criteria set out above should be able to consent for themselves regardless of age and whether they have reached majority.
